# Population structure and phylogeography reveal pathways of colonization by a migratory marine reptile (*Chelonia mydas*) in the central and eastern Pacific

**DOI:** 10.1002/ece3.1269

**Published:** 2014-10-25

**Authors:** Peter H Dutton, Michael P Jensen, Amy Frey, Erin LaCasella, George H Balazs, Patricia Zárate, Omar Chassin-Noria, Adriana Laura Sarti-Martinez, Elizabeth Velez

**Affiliations:** 1 Marine Mammal & Turtle Division, Southwest Fisheries Science Center, National Marine Fisheries Service, National Oceanic and Atmospheric Administration8901 La Jolla Shores Drive, La Jolla, California, 92037; 2 Pacific Islands Fisheries Science Center, National Marine Fisheries Service, National Oceanic and Atmospheric Administration1845 Wasp Blvd., Honolulu, Hawaii, 96818; 3 Archie Carr Center for Sea Turtle Research and Department of Biology, University of FloridaPO Box 118525, Gainesville, Florida, 32611; 4 Facultad de Biología, Centro Multidisciplinario de Estudios en Biotecnología- UMSNHMorelia, Michoacán, 58030, México; 5 Dirección de Especies Prioritarias para la Conservación, CONANPCamino al Ajusco 200, 2° piso Ala Sur, Col. Jardines en la Montaña, México, DF, 14210, México; 6 Kelonian Conservation SocietyHeredia, Costa Rica

**Keywords:** *Chelonia mydas*, genetic stock structure, marine turtles, mtDNA, phylogeography

## Abstract

Climate, behavior, ecology, and oceanography shape patterns of biodiversity in marine faunas in the absence of obvious geographic barriers. Marine turtles are an example of highly migratory creatures with deep evolutionary lineages and complex life histories that span both terrestrial and marine environments. Previous studies have focused on the deep isolation of evolutionary lineages (>3 mya) through vicariance; however, little attention has been given to the pathways of colonization of the eastern Pacific and the processes that have shaped diversity within the most recent evolutionary time. We sequenced 770 bp of the mtDNA control region to examine the stock structure and phylogeography of 545 green turtles from eight different rookeries in the central and eastern Pacific. We found significant differentiation between the geographically separated nesting populations and identified five distinct stocks (*F*_ST_ = 0.08–0.44, *P* < 0.005). Central and eastern Pacific *Chelonia mydas* form a monophyletic group containing 3 subclades, with Hawaii more closely related to the eastern Pacific than western Pacific populations. The split between sampled central/eastern and western Pacific haplotypes was estimated at around 0.34 mya, suggesting that the Pacific region west of Hawaii has been a more formidable barrier to gene flow in *C. mydas* than the East Pacific Barrier. Our results suggest that the eastern Pacific was colonized from the western Pacific via the Central North Pacific and that the Revillagigedos Islands provided a stepping-stone for radiation of green turtles from the Hawaiian Archipelago to the eastern Pacific. Our results fit with a broader paradigm that has been described for marine biodiversity, where oceanic islands, such as Hawaii and Revillagigedo, rather than being peripheral evolutionary “graveyards”, serve as sources and recipients of diversity and provide a mechanism for further radiation.

## Introduction

Climate, behavior, ecology, and oceanography have been shown to shape the evolution of patterns of biodiversity in marine faunas in the absence of obvious geographic barriers that tend to be prominent in the terrestrial realm (Dawson and Hamner 2008). For highly migratory marine vertebrates, dispersal and gene flow (between populations) is influenced by behavior and ecology (Baker et al. 1998; Foote et al. 2011; Ansmann et al. 2012; Hoffman and Forcada 2012) as opposed to corals, mollusks, crustaceans, and fishes where larval dispersal plays a prominent role (Crandall et al. 2012). Bowen et al. (2013) recently proposed a new biogeographic model called “Biodiversity Feedback” that integrates previous models for marine speciation with biodiversity hotspots acting as both Centers of Speciation and Centers of Accumulation and/or Overlap and showed that peripheral habitats such as the oceanic Hawaiian archipelago are not evolutionary graveyards, but instead can export biodiversity (Bowen et al. 2013). Although focusing on speciation processes and tropical reef ecosystems, biodiversty feedback provides a useful framework for examining phylogeographic diversity at the population level for widely distributed species. Marine turtles are an example of globally distributed, highly migratory species with deep evolutionary lineages and complex life histories that span both terrestrial and marine environments (Jensen et al. 2013), unlike corals, fish, and cetaceans that live entirely in the sea. The green turtle, *Chelonia mydas*, has the most contiguous distribution of the 7 marine turtle species (Wallace et al. 2010). Geography and climate appear to have shaped diversity in sea turtles with the onset of glacial cycles, the appearance of the Panama Isthmus land barrier separating the Atlantic and eastern Pacific (between 5 and 2.5 mya; Farrell et al. 1995), the cooling of southern ocean waters in the mid to late Miocene (between 17 and 6 mya; Rögl 1998), and upwelling of cold water off southern Africa creating an oceanographic barrier between the Atlantic and Indian Ocean (Shannon 1985). Recent warm temperatures during interglacial periods allowed a reverse invasion from the Atlantic and back into the Indian Ocean, although the scale and timing of this connectivity remains unknown (Formia et al. 2006; Bourjea et al. 2007; Bowen and Karl 2007). Today, it appears that green turtles within an ocean basin are effectively isolated from populations in the other basins. Phylogeographic studies have focused on the establishment of the Panama Isthmus land barrier leading to the deeper isolation of two matriarchal Atlantic and Pacific lineages (3–7 mya) (Bowen et al. 1992; Dutton 1996; Bowen and Karl 2007; Naro-Maciel et al. 2008; Duchene et al. 2012); however, little attention has been given to the pathways of colonization of the eastern Pacific and the processes that have shaped diversity within the most recent millennium.

Marine turtle life history involves adult migrations from feeding grounds to distant breeding sites, as well as ontogenetic changes that affect the distribution of juveniles across a variety of marine habitats (Musick and Limpus 1997). After emerging from nests laid on tropical (and subtropical) beaches, hatchlings enter the water and are dispersed by ocean currents, generally spending the first years of life in a pelagic phase before transitioning to a variety of coastal and neritic developmental and foraging habitats where they often take up long-term residence (Musick and Limpus 1997). Because of this complex life history, defining stock boundaries remains challenging, yet understanding the connectivity among the breeding populations and the foraging and migratory populations across various geographic scales is important for the conservation of these endangered and threatened species. Natal homing to breeding sites is now well established as the primary mechanism that restricts dispersal of genes in marine turtles (Allard et al. 1994), but variation in the precision and rigidity of natal homing among nesting populations of the same species in different regions affects the degree and geographic scale of population structure (Jensen et al. 2013).

**Figure 1:**
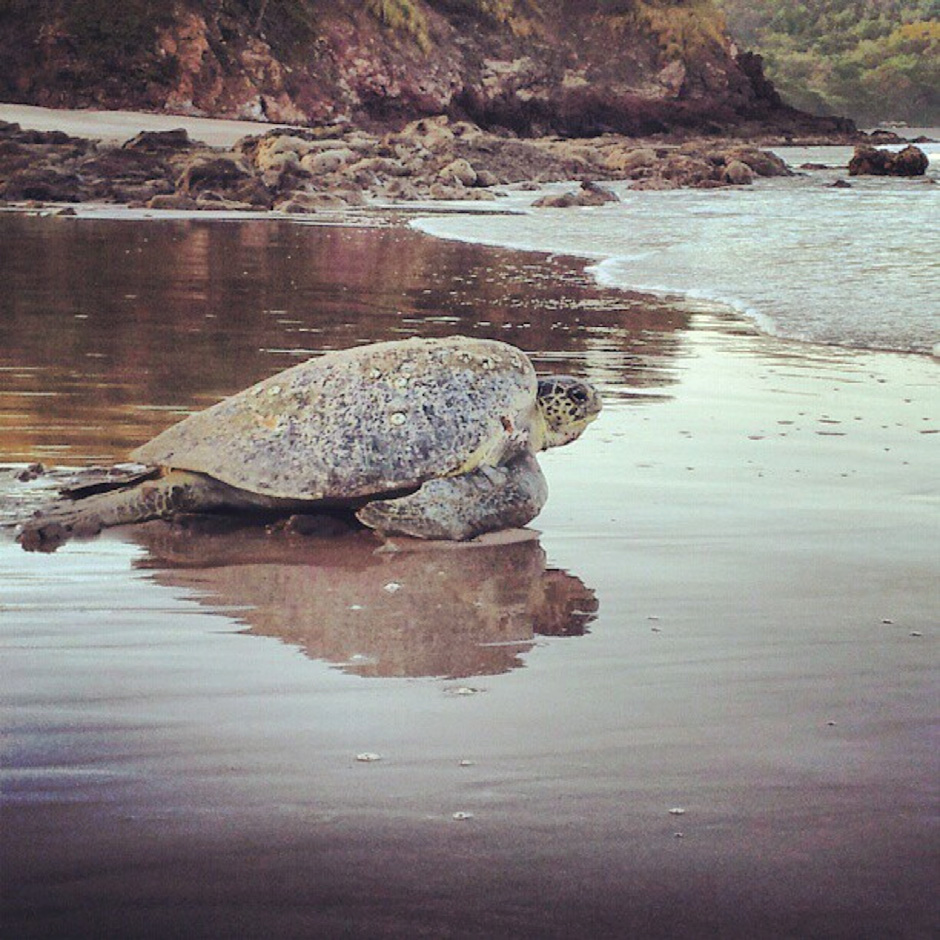
Green turtle returning to the ocean after nesting at Nombre de Jesús beach, Guanacaste, Costa Rica (photo by Billy Leal).

Green turtles nest at numerous locations on beaches scattered throughout the Pacific. Only one major rookery occurs in the central North Pacific, on French Frigate Shoals (FFS), a remote Atoll in the Northwest Hawaiian Archipelago. This population has been increasing steadily over the past 30 years in response to long-term conservation efforts (Balazs and Chaloupka 2004, 2006). Minor nesting also occurs on some of the other Hawaiian Atolls, notably Laysan Island, approximately 600 km northwest of FFS. In the eastern Pacific, major nesting populations occur at Maruáta and Colola beaches in the state of Michoacán in Mexico and in the Galápagos Islands, Ecuador. The Mexican rookery received much attention after dramatic population declines in the 1970's and is showing signs of recovery in recent years (Alvarado-Díaz and Figueroa 1990; Delgado-Trejo and Alvarado-Diaz 2012). Less is known about the Galápagos nesting populations; however, recent surveys indicate that large numbers of *C. mydas* still nest there and that rookeries at the Galápagos have not experienced the population declines that have characterized other eastern Pacific *C. mydas* rookeries (Zárate 2012). Two other major rookeries have been recently discovered in the eastern Pacific: expeditions to the Revillagigedo Archipelago off the Mexican coast in 1999 and 2000 revealed significant *C. mydas* nesting on Socorro and Clarion Islands (Dutton et al. 2008; Holroyd and Trefry 2010), and in 2004, a beach in Guanacaste Province in Costa Rica was found to host the most significant *C. mydas* rookery in Pacific central America (Blanco et al. 2012) (Fig. 1).

Genetic studies of maternally inherited mitochondrial DNA (mtDNA) have been useful in understanding the population structure and reproductive behavior of these highly migratory marine animals (Bowen et al. 1992; FitzSimmons et al. 1997; Formia et al. 2006; Dutton et al. 2008), and in demonstrating the existence of distinguishable stocks for management purposes, or management units (MUs) (Moritz 1994; Bowen and Karl 2007). Dethmers et al. (2006, 2010) characterized stock boundaries for *C. mydas* populations in the western and Indo-Pacific based on extensive sampling of 27 rookeries and found that most rookeries located within 500 km of each other were genetically indistinguishable. However, efforts to determine stock structure for *C. mydas* nesting populations elsewhere in the Pacific have been more limited. Chassin-Noria et al. (2004) characterized a single rookery in Michoacán, Mexico using 400-bp sequences of the mtDNA control region. More recently, Dutton et al. (2008) demonstrated that *C. mydas* foraging in the Hawaiian Archipelago comprise a single genetic stock belonging to the nesting population in FFS, using baseline data of potential source rookeries from the central and eastern Pacific (FFS, Galápagos, and Mexico), and Amorocho et al. (2012) estimated the stock composition of juvenile *C. mydas* at a feeding ground off Colombia using the same rookery baseline data set. However, a comprehensive rookery stock structure analysis for eastern and central Pacific *C. mydas* has not been performed. This not only hinders the development of effective management strategies for the nesting habitats, but also limits the ability to carry out accurate mixed stock analysis (MSA) to trace back the natal origin of turtles sampled at foraging areas and migratory corridors, important for assessing stock-specific threats (Jensen et al. 2013).

While the mtDNA control region has been a useful marker for detecting stock structure, previous studies have shown that common and widespread haplotypes make it harder to detect fine scale structure for many marine turtle species (Dutton et al. 1999; Dethmers et al. 2006; Jensen et al. 2013). Longer sequences might have additional variation to help resolve those common haplotypes and provide increased resolution to stock structure (LeRoux et al. 2012; Shamblin et al. 2012; Dutton et al. 2013; Jensen et al. 2013). In some cases, this lack of resolution is particularly problematic for precise stock assignment of *C. mydas* at foraging grounds and in fisheries bycatch in the region. For example, CmP4 (a 384-bp mtDNA Control Region haplotype in *C. mydas*) is common and widespread at foraging grounds and rookeries in the eastern Pacific (Dutton et al. 2008; Amorocho et al. 2012).

In this study, we use mtDNA markers to identify the stock structure and phylogeography of eight key rookeries in the central and eastern Pacific and examine potential pathways of colonization of this region within the most recent evolutionary time. We build on previous work by (1) reanalyzing available samples using longer 770-bp sequences; (2) substantially increasing the sample size for previously analyzed rookeries, and (3) including samples from new rookeries in Costa Rica and the Galápagos Islands for a comprehensive coverage of the nesting distribution. Finally, we consider implications of our findings for conservation of this threatened species.

## Methods

### Sample collection

**Figure 2:**
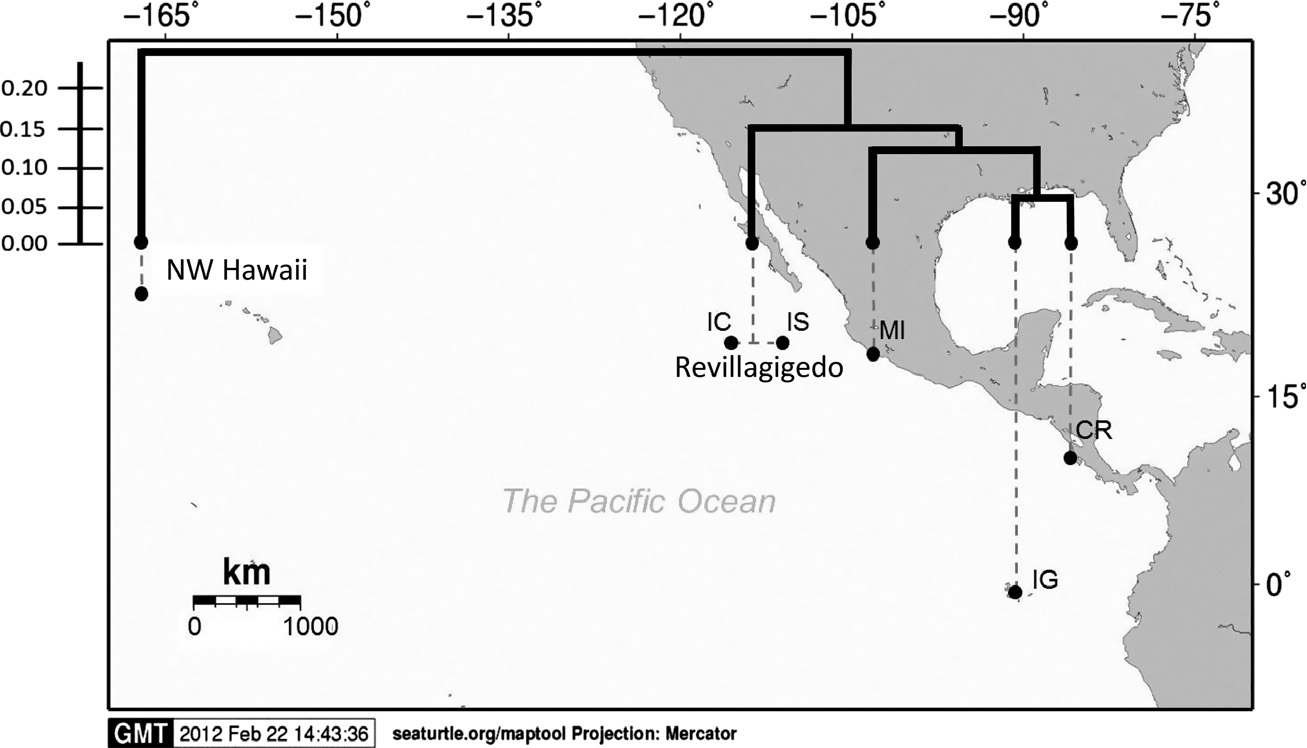
Map depicting the sampling locations of *Chelonia mydas* rookeries at NW Hawaii (French Frigate Shoals and Laysan), Revillagigedo (IC, Isla Clarion and IS, Isla Socorro), MI (Michoacán), CR (Costa Rica), and IG (Galápagos Islands including two sites at Las Bachas and Las Salinas). The genetic relationship between stocks is shown with an UPGMA tree showing pairwise F_ST_ values.

A total of 545 samples were collected from eight *C. mydas* nesting populations in the central and eastern Pacific between 1990 and 2009. Tissue types collected include tissue salvaged from dead hatchlings and skin biopsies or blood collected from nesting females (Dutton 1996). Skin samples were stored in vials in 70% ethanol or a saturated salt solution. Whole blood or packed red blood cells drawn from centrifuged samples were stored in 2 mL cryovials (Dutton 1996). All samples are stored at −20°C at the Southwest Fisheries Science Center Marine Mammal and Turtle Molecular Research Sample Collection (La Jolla, CA, USA). In general, for nesting turtles, a blood or skin sample was collected during nesting events. The data set from the nesting sites included samples from five regions (1) northwest Hawaii (190 from French Frigate Shoals and 12 samples from Laysan Island), (2) 120 samples from Colola beach in Michoacán, Mexico, (3) 77 samples from the Revillagigedo Islands, Mexico (58 from Clarion and 19 from Socorro Islands), (4) 20 samples from Nombre de Jesús beach in Guanacaste, Costa Rica, and (5) 126 samples from the Galápagos Islands (45 from Las Bachas and 81 from Las Salinas beaches on Santa Cruz and Baltra Island, respectively), (Fig. 2, Table 1).

**Table 1:** Frequencies of green turtle (*Chelonia mydas*) mtDNA haplotypes among eight central and eastern Pacific rookeries based on ca. 770 bp

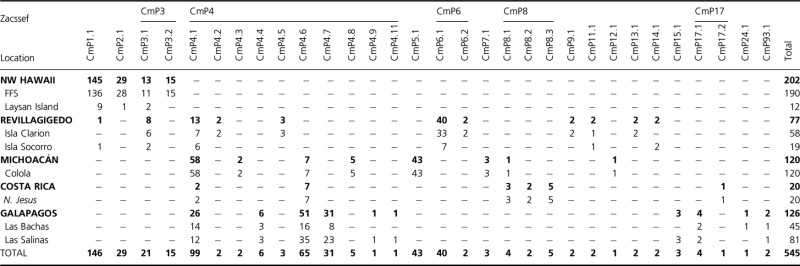

### Laboratory analysis

Genomic DNA was isolated from samples of tissue and blood using one of the following standard extraction techniques: Phenol/chloroform (Sambrook et al. 1989), sodium chloride (Miller 1988), a modified DNEasy® Qiagen extraction kit (Qiagen, Valencia, CA), or a Corbett CAS-1200 extraction robot (Corbett Robotics, San Francisco, CA; Dutton et al. 2008). Primers LCM15382 and H950 g were used to amplify an ∼889-bp fragment at the 5′ end of the control region of the mitochondrial genome using polymerase chain reaction (PCR) methodology (Abreu-Grobois et al. 2006; Dutton et al. 2007) A 25 *μ*L PCR reaction was used with the following composition: 18 *μ*L purified H_2_O, 2.5 *μ*L of 10× Mg buffer, 1.5 *μ*L DNTPs, 0.75 *μ*L of each primer, 0.5 *μ*L of Taq polymerase, and 1 *μ*L (20–50 ng) of template DNA. PCRs were run with the following profile: initial DNA denaturation at 94°C for 2 min, followed by 36 cycles of (1) DNA denaturation at 94°C for 50 sec, (2) annealing of primers at 52°C for 50 sec, and (3) extension of primers at 72°C for 1 min, concluding with a final extension of primers at 72°C for 5 min. Negative controls were included in each PCR to detect contamination. PCR products were purified by combining 5 *μ*L of product with 2 *μ*L of an exonuclease I and Shrimp Alkaline Phosphatase solution. PCR products were cycle sequenced in both directions using a 12-*μ*L reaction consisting of a 1:1 buffered version of the ABI® Big Dye Terminator v 3.1. Labeled extension products were purified using an ethanol precipitation process. Products were analyzed with an Applied Biosystems® model 3130 automated DNA sequencer (Applied Biosystems, Foster City, CA). All sequences were trimmed to ∼770 bp for further analyses as this region contains high-quality sequence.

### Statistical analysis

Sequences were analyzed using the program SeqScape v2.5 (Applied Biosystems). Each sequence was reviewed manually for uncalled and miscalled bases, and all variable positions were confirmed by comparing sequences from the forward and reverse strands. We assigned haplotypes by comparing aligned sequences against a local reference library of approximately 770-bp haplotype sequences using Geneious Pro 6.0.2 (Drummond et al. 2011) as well as searching the database on GenBank (http://www.ncbi.nlm.nih.gov). We standardized nomenclature of haplotypes based on these 770-bp alignments, assigning the CmP prefix to numerically sequential names based on the original 384-bp alignments (Dutton et al. 2008) with a sequential numeric suffix to indicate a variant resulting from polymorphism in the additional 386-bp region flanking the old shorter sequence (e.g., CmP4.1, CmP4.2 etc.). Unique sequences were then aligned with the CLUSTALW algorithm implemented in Geneious Pro 5.6.3 (Drummond et al. 2011). The alignment of each mtDNA segment was checked and edited by eye separately. Haplotype (*h*) and nucleotide (*π*) diversity were calculated for each rookery using Arlequin v 3.5.1.2 (Excoffier and Lischer 2010). Haplotype diversity was estimated based on Nei (1987), and nucleotide diversity was calculated assuming the model of Tamura and Nei (1993). We tested for population structure by conducting analysis of molecular variance (AMOVA) (Excoffier et al. 1992) using both F_ST_ and Φ_ST_ measures in Arlequin. Significance values for AMOVA were obtained from 10,000 permutations. In the AMOVA, rookeries were grouped by their identified MUs. Both conventional *F*_ST_ and sequence-based Φ_ST_ distance measures as well as the exact test were used to calculate within- and among-population diversity. The *F*_ST_ and Φ_ST_ pairwise comparisons of population differentiation were conducted with 10,000 permutations and the exact test with 10,000 permutations and 10,000 dememorization steps (Raymond and Rousset 1995). The best substitution model of sequence evolution (TrN+I, gamma shape = 0.8480; Tamura and Nei 1993) was determined by jModelTest 0.1.1 (Guindon and Gascuel 2003; Posada 2008) and the corrected Akaike information criterion. To test the assumption of a molecular clock, a maximum-likelihood phylogenetic tree was reconstructed using MEGA v5.05 under the TrN + I mutation model. In this test, the null hypothesis of a molecular clock could not be rejected (*P* > 0.05) for the eastern Pacific data set. We constructed statistical parsimony haplotype networks (Tempelton et al. 1992; Posada and Crandall 2001) to depict patterns of genetic variation between the haplotypes using the software TCS v.1.21 (Clement et al. 2000). We also used MEGA v5.05 to construct a UPGMA tree using pairwise F_ST_ values to depict the relationship among sampled rookeries and to evaluate their relationship to rookeries in the western Pacific using data from Dutton et al. (2014). The data set was assessed for the relationships among haplotypes using the strict phylogenetic approach implemented in BEAST 1.6.0 (Drummond and Rambaut 2007). The tree was rooted using *Natator depressus* (GenBank acc. no. U40662) as the out-group. Divergence dates were estimated with BEAST v 1.6.1 (Drummond and Rambaut 2007; http://beast.bio.ed.ac.uk.). A strict molecular clock of 0.01751 subs/site/mya was used based on rates calculated for *C. mydas* (Formia et al. 2006). The analysis was performed using a random starting tree (tree prior speciation: yule process) running three independent chains and 50 million generations, chains were sampled every 1000 generations with a burn-in of 2 million generations. Results were analyzed with the program Tracer v. 1.5 (Drummond and Rambaut 2007).

## Results

### Sequence diversity

Analysis of the 545 sequences identified a total of 30 polymorphic positions that defined a total of 31 haplotypes (GenBank accession numbers KC306643–KC306671, FJ917195, and KC771270; Appendix Table A1). There were 26 substitutions (25 transitions and one transversion). Four indels were present of which one was an eight base indel (found only in CmP93.1 from the Galápagos) (Table 1). Ten of the haplotypes were variants of the 384-bp CmP4 haplotype (Dutton et al. 2008) that contained polymorphism in the additional sequence portion. CmP3, 6, 8, and 17 also contained variants defined by additional polymorphism in the new portion (Appendix Table A1). The large rookery at NW Hawaii was comprised of only four haplotypes dominated by CmP1.1 (72%) and only shared two haplotypes with Revillagigedo at low frequency. The haplotype CmP4.1 was found at intermediate frequency (10–48%) at all rookeries except Hawaii and generally characterized the eastern Pacific rookeries. Combined, the two Revillagigedo rookeries were characterized by a large number of haplotypes (*n* = 11) of which eight were endemic. Moreover, they were also the only rookeries to share one or more haplotypes with all other rookeries in the eastern Pacific. The rookery in Michoacán also had a high number of haplotypes (*n* = 8) with five endemic ones. The rookery in Costa Rica was characterized by the haplotypes CmP8.3 (25%), CmP8.1 (15%), and CmP8.2 (10%) although a total of six haplotypes were identified with three being endemic. Finally, the Galápagos Islands rookeries had a high number of haplotypes (*n* = 10) including eight endemic ones (Table 1). For all rookeries, haplotype (gene) diversity was low to moderate ranging from *h* = 0.4567 at Hawaiian rookeries to 0.8105 at rookeries in the Galápagos, while nucleotide diversity was low, ranging from *π* = 0.0014 in the Galápagos to *π* = 0.0034 at the Costa Rican rookery (Table 2).

**Table 2:** Genetic diversity parameters for the eight green turtle rookeries and five genetic stocks identified in the central and eastern Pacific based on ca. 770 bp mtDNA control region sequences. The table shows sample size (*n*), number of haplotypes (*H*), nucleotide (π) and haplotype (*h*) diversity, and their standard deviation (SD)

Location	*n*	*H*	*π*	SD	*h*	SD
NW Hawaii	202	4	0.00200	0.00133	0.4567	0.0388
FFS	190	4	0.00204	0.00135	0.4588	0.0398
Laysan Island	12	3	0.00179	0.00133	0.4394	0.1581
Revillagigedo	77	11	0.00184	0.00126	0.6941	0.0499
Isla Clarion	58	9	0.00185	0.00127	0.6546	0.0649
Isla Socorro	19	6	0.00181	0.00130	0.7778	0.0640
Michoacán	120	8	0.00227	0.00147	0.6371	0.0285
Colola	120	8	0.00227	0.00147	0.6371	0.0285
Costa Rica	20	5	0.00238	0.00159	0.7316	0.0644
N. Jesus	20	5	0.00238	0.00159	0.7316	0.0644
Galápagos	126	10	0.00148	0.00107	0.7346	0.0234
Las Bachas	45	7	0.00188	0.00129	0.7545	0.0356
Las Salinas	81	9	0.00182	0.00125	0.7157	0.0330
Overall	545					

### Population structure

The pairwise test showed that Laysan was not significantly different from FFS (*F*_ST_ = −0.02719; *P* = 0.759) in the NW Hawaiian islands; the two islands representing Revillagigedo Islands (Socorro and Clarion) were not significantly different (*F*_ST_ = 0.03730; *P* = 0.073), and finally Las Salinas and Las Bachas (Galápagos) were also not significantly different (*F*_ST_ = −0.01486; *P* = 0.120). Samples for these nonsignificant comparisons were pooled to represent single genetic stocks (or MUs) in subsequent analyses. Further pairwise comparisons were all highly significant identifying a total of five genetically independent stocks (or MUs) (Table 3, Fig. 2). The AMOVAs among MUs supported the pattern of strong genetic structure (*P* < 0.001). Overall, the proportion of variation found across the five east-ern Pacific MUs was greater within (*F*_ST_ = 54.85%; Φ_ST_ = 63.74%) vs. among (*F*_ST_ = 45.15%, Φ_ST_ = 36.26%) MUs (Table 3).

**Table 3:** Pairwise Φ_ST_, *F*_ST_ and Fisher's exact test for population differentiation (all below the diagonal) for the five genetic stocks identified in the central and eastern Pacific. All comparisons were highly significant (*P* < 0.005). Approximate in-water distance between the rookeries are given above the diagonal

	NW Hawaii	Revillagigedo	Michoac	Costa Rica	Galápagos
NW Hawaii		>5300 km	>6500 km	>8500 km	>8500 km
Revillagigedo	0.44057 ** **		>800 km	>3200 km	>3300 km
F_ST_					
Exact test					
Michoacán	0.46419 ** **	0.27724 ** **		>2000 km	>2400 km
F_ST_					
Exact test					
Costa Rica	0.46199 ** **	0.27861 ** **	0.27415 ** **		>1200 km
F_ST_					
Exact test					
Galápagos	0.41834 ** **	0.25845 ** **	0.21733 ** **	0.12464 ** **	
F_ST_					
Exact test					

### Phylogenetic analysis and demographic history

**Figure 3:**
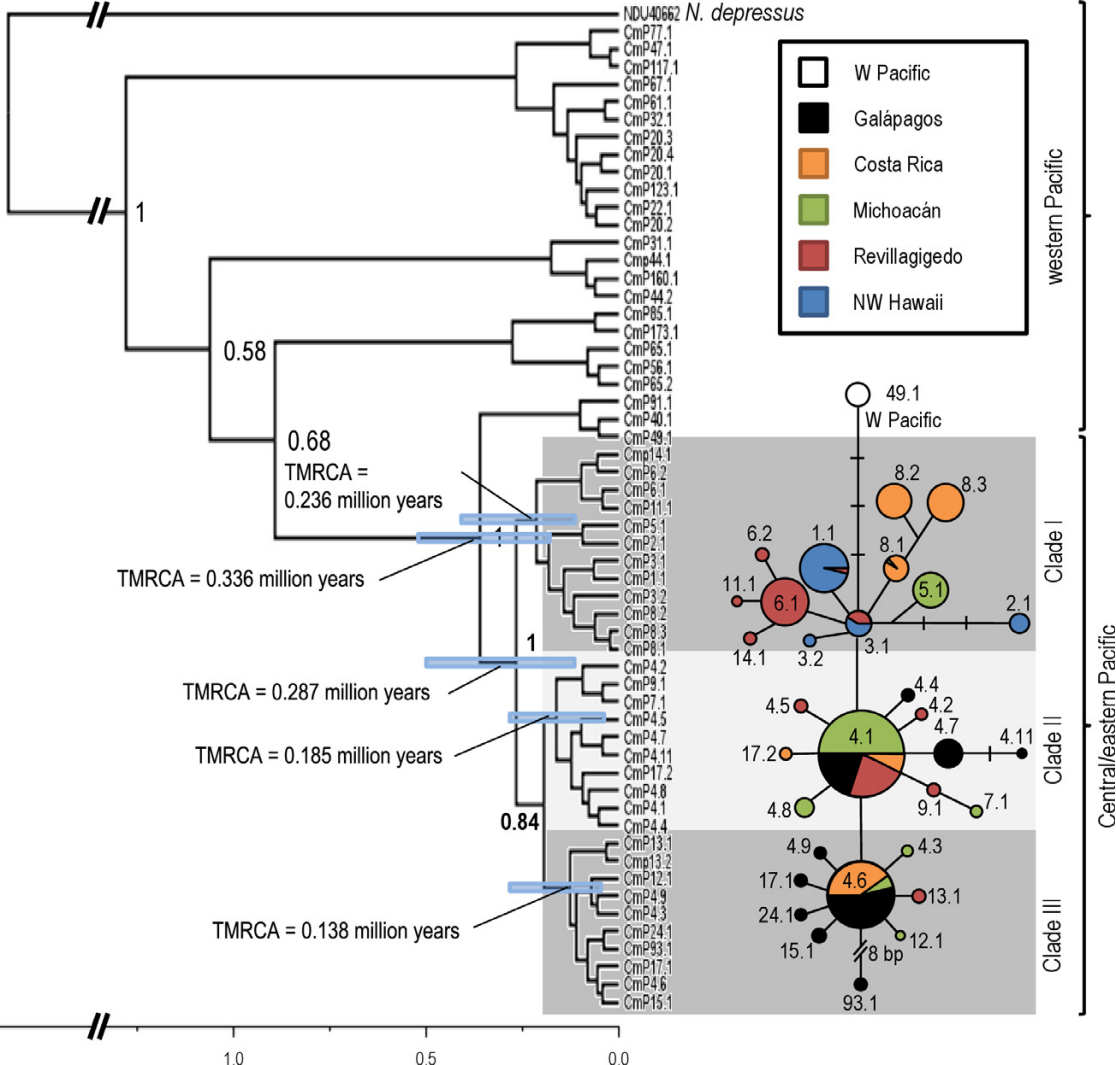
Phylogeny of eastern and central Pacific *Chelonia mydas* haplotypes showing divergence times (TMRCA million years before present, *x*-axis) calculated using the program BEAST for the coalescents among 770-bp green turtle mtDNA control region lineages from the central and eastern Pacific. Mean highest posterior density (HPD) values estimated for tree nodes are indicated together with their corresponding 95% HPD intervals (blue shaded horizontal bars). Relationships between haplotypes are shown by the most parsimonious median-joining network, indicating groupings of 3 major clades. Number of mutations between haplotypes is illustrated by dashes in connecting lines and correspond to data in Table 1. The position of an 8-bp insertion is indicated. The size of the circles is approximately proportional to haplotype frequency in the overall sample set. Colors denote the locations where individual haplotypes were detected and the proportions of shared haplotypes that were distributed among different rookeries.

The network of haplotype relationships in the central/eastern Pacific is characterized by star-shaped phylogenies clustered around common haplotypes with a somewhat geographically partitioned distribution, although a pattern of multiple colonization events is evident across most rookeries (Fig. 3). The software suggested that CmP4.1 was the ancestral haplotype; however, the western Pacific haplotype CmP49.1 was identified as the most closely related to the eastern Pacific and connected to the Hawaiian haplotype CmP3.1. This latter haplotype, while found at low frequency is centrally located in the network, suggesting that it might be ancestral to all other eastern Pacific haplotypes, with CmP4.1 being central to more recent colonization of the eastern Pacific (Fig. 3). Overall, sequence divergence for the region was very low and ranged from 0.13 to 0.79%.

**Figure 4:**
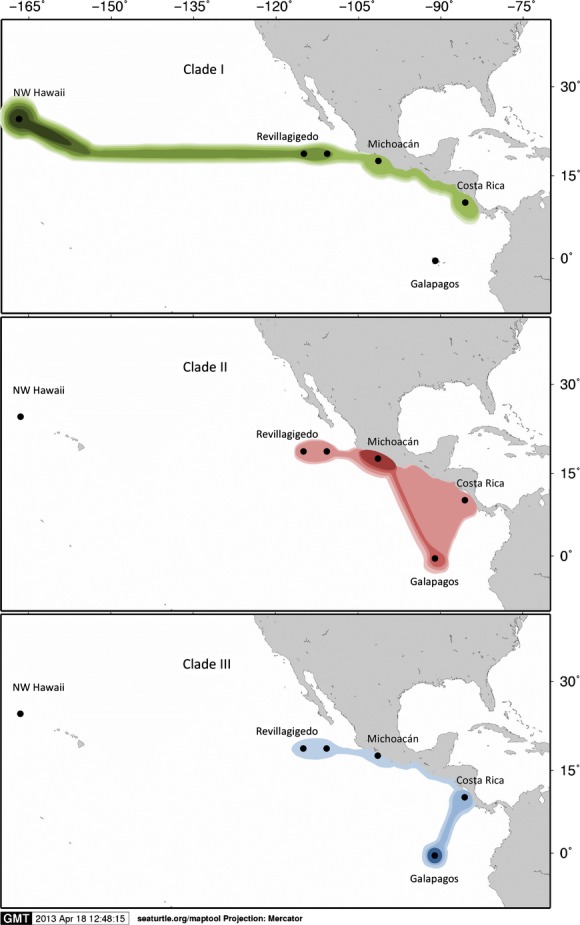
The geographic distribution of three clades (Clade I, II and III) among central (NW Hawaii) and eastern Pacific (Revillagigedo, Michoacán, Costa Rica and Galápagos) *Chelonia mydas* rookeries. Darker shade coloring indicates greater relative presence of haplotypes from a clade.

The Bayesian phylogenetic tree indicated that haplotypes from the eastern Pacific rookeries form a distinct monophyletic group with robust support for the split between the central/eastern and western Pacific with a Bayesian posterior probabilities of 1.0 (Fig. 3). While most of the internal phylogeny of the central/eastern Pacific exhibited very short branches with low support, three distinct subclades were evident within the central/eastern Pacific (posterior probabilities of 1.0 and 0.84). The split between sampled central/eastern and western Pacific haplotypes was estimated at 0.336 mya (95% HPD: 0.178–0.505 mya). The mean time to the most recent common ancestor (TMRCA) of all central/eastern Pacific haplotypes was estimated at 0.287 mya (95% HPD: 0.129–0.493 mya), and the TMRCA of the central/eastern Pacific subclade I, II, and III was 0.236 mya (95% HPD: 0.122–0.403 mya), 0.185 mya (95% HPD: 0.065–0.285 mya), and 0.138 mya (95% HPD: 0.068–0.227 mya), respectively (Fig. 3). The geographic distribution of the three clades shows that the oldest (clade I) is the only clade present in the NW Hawaii (100%), common in the Revillagigedo Islands (∼70%), and less so in Michoacán (∼37%) and Cost Rica (∼50%), while it is not found in the Galápagos Islands. The second oldest clade (Clade II) is not found in the NW Hawaii but is common in Revillagigedo Islands (∼30%), Michoacán (∼55%), and the Galápagos Islands (∼55%), but less common in Costa Rica (∼15%). Finally, the youngest clade (clade III) is not found in the NW Hawaii, is very rare in Revillagigedo Islands (∼1%), Michoacán (∼8%), and common in Costa Rica (∼35%) and the Galápagos Islands (∼50%) (Fig. 4).

## Discussion

### Population structure

Our finding that the main rookeries in the central and eastern Pacific are strongly differentiated indicates that the nesting female populations are demographically isolated. The F_ST_ levels are similar to other marine turtle studies when considering the scale involved. The rookeries that were grouped together based on nonsignificant *F*_ST_ values (two in Hawaii, two in Revillagigedo, and two in the Galápagos) were all located 5–600 km from each other, while the individual genetic stocks were all located 800–8500 km from each other. Dethmers et al. (2006) suggested a rule of thumb for *C. mydas* across the Indo-Pacific that rookeries separated by more than 500 km could generally be considered independent stocks. The observed F_ST_ values are likely high because most of the rookeries are oceanic islands, which tend to strengthen isolation and differentiation compared with coastal rookeries. For instance, loggerheads in the western Atlantic where nesting is dispersed over a large area of continental coastline, tend to have weaker differentiation between proximate rookeries (Shamblin et al. 2011). We found no evidence of differentiation between Laysan and FFS, and despite the small sample size for Laysan, the presence of the same three haplotypes at similar frequencies as at FFS indicates that this negative result is probably valid. A previous study showed that juvenile and adult populations foraging around the Hawaiian Islands form one genetic stock (Dutton et al. 2008). Our results reinforce the conclusion that *C. mydas* nesting sites in the NW Hawaiian archipelago are part of a single population. Other minor nesting sites in NW archipelago (e.g., Lisianski, Pearl and Hermes, and Midway atolls) should be sampled to confirm this. The predictions of sea level rise have led to speculation that some of the nesting sites may become inundated causing changes in the distribution of nesting as turtles seek out other beaches suitable for nesting (Baker et al. 2006). For instance, in the 1960's, Whaleskate Island was the second largest nesting beach for *C. mydas* in FFS. The low island slowly eroded and by the late 1990s, it was completely submerged (Baker et al. 2006). These scenarios are likely to occur on a local regional scale as sea levels increase, and the increase in sporadic nesting that has been observed on the main populated Hawaiian Islands (Molokai, Hawaii, Maui, and Oahu) in recent years may represent new colonization by migrants from the NW Hawaiian islands (Frey et al. 2013).

While the sampled rookery at FFS contains more than 90% of the nesting in the NW Hawaiian islands, the sampling along central America and the Galápagos is more fragmented and smaller unsampled locations are bound to exist (e.g., Ecuador; M. Peña, pers. comm.). There is no documented historic nesting along Baja California, with the exception of a few recent reports of *C. mydas* nests identified near La Paz (L. Sarti-Martinez, pers. obs).

It is interesting that the Costa Rica rookery is strongly differentiated from the other mainland rookery in Mexico, while being more closely related to the Galápagos rookery. The Costa Rica nesting population was only recently discovered, and while it may be the largest *C. mydas* nesting aggregation in Central America, it appears to be relatively small compared with Michoacán and Galápagos. The presence of old endemic haplotypes suggests that this area was not recently colonized but shows signs of a long-term stable population with a mix of divergent haplotypes. There may be mixing of the Galápagos, Michoacán, and Costa Rica animals on foraging grounds (Green 1984; Alvarado-Díaz and Figueroa 1992; Seminoff et al. 2008; Amorocho et al. 2012), but our results suggest that the nesting populations are segregated by female natal homing behavior. The relatively greater connectivity between Galápagos and Costa Rica may indicate historic migration between these regions. Tagging studies have documented migration of Galápagos nesters to distant foraging areas off Central America (Green 1984), but not farther north to Mexico, suggesting potential for historic colonization by dispersers. Any such dispersal would have happened a long time ago (>130,000 ya).

While our study shows strong genetic divergence based on mtDNA, we cannot be completely sure that these rookeries do not exchange genes, as any male-mediated gene flow would remain undetected by our mtDNA markers, and nuclear DNA studies are needed to detect it. However, Roden et al. (2013) in a parallel study used SNPs and microsatellites to investigate nuclear stock structure and found significant differentiation between the Michoacán, Galápagos, and NW Hawaii rookeries (Revillagigedo and Costa Rica were not included) consistent with our mtDNA results and suggesting that male-mediated gene flow between regional nesting stocks is more limited than previously believed.

### Phylogeography and diversity

We use the molecular clock approach to study the origin of *C. mydas* and subsequent evolutionary change in space and time by relating substitution rates to divergence times. It is important to stress that the accuracy of the molecular clock has long been subject to controversy, and numerous papers have addressed problems associated with estimating divergence times, particularly for a species like marine turtles that have a general lack of reliable calibration points to estimate precise clock rates (see Warnock et al. 2012). However, while our molecular clock and subsequent divergence times should be interpreted with caution they provide interesting information about the relative timing of colonization and the broad timescale, that is, at play.

From both the phylogenetic tree and the haplotype network, it is evident that haplotypes from the eastern and central Pacific *C. mydas* form a monophyletic group and that Hawaii is more closely related to eastern Pacific green populations than populations in the western Pacific. The haplotype CmP49.1 (C3 for the 386 bp used in Dethmers et al. 2006), which is the closest related haplotype to the central/eastern Pacific is the most common and widespread haplotype across the Indo-Pacific (Dethmers et al. 2006; Dutton et al. 2014). To date, none of the Hawaiian, or any other eastern Pacific haplotypes have been found at western Pacific rookeries (Dethmers et al. 2006; Cheng et al. 2008; Nishizawa et al. 2013; Dutton et al. 2014). This suggests a phylogeographic break or barrier (coalescing around 0.336 mya) west of the Hawaiian chain that separates the central and eastern rookeries from the other Pacific regions. The East Pacific Barrier (EPB), the expanse of deep ocean separating the Eastern Tropical Pacific (ETP) from Central Pacific (CP) (Ekman 1953) is a well-known barrier that has been shown to limit dispersal and explain phylogeographic patterns (and patterns of speciation) for larval dispersers the in eastern and central Pacific (Vermeij 1987; Craig et al. 2007; Duda & Lessios 2009; Chow et al. 2011; Baums et al. 2012). Our findings show that the West Pacific appears to be a more formidable barrier to gene flow (colonization) in *C. mydas* on an ocean scale than the EPB has been for other marine taxa and suggests that colonization of nesting beaches by vagrant reproductive females from the western Pacific is an extremely rare event, despite the presence of western Pacific juveniles in eastern Pacific waters (Amorocho et al. 2012).

The patterns of genetic diversity found in this study further contrast with other *C. mydas* populations as well as other species such as *Caretta caretta* and *Eretmochelys imbricata* in other broad oceanic regions (Atlantic-Caribbean, Indo-Pacific) that typically contain individuals from two or more highly divergent haplogroups indicating older colonization events, punctuated by divergence and secondary contact (Bowen et al. 1994; Dethmers et al. 2006; Bourjea et al. 2007; LeRoux et al. 2012). Our results (Fig. 3) suggest that CmP4.1 is an ancestral regional haplotype for the eastern Pacific while CmP3.1 is ancestral in NW Hawaii. CmP3.1 is closely related to the haplotype CmP49.1 which is common and widespread throughout the western Pacific and Indian Ocean and likely represents the link to colonization into the eastern Pacific from the western Pacific via NW Hawaii. Older alleles tend to be more central in haplotype networks, have more mutational connections and are often present in higher frequencies. Ancestral haplotypes also tend to be widespread across subdivided populations (Takahata 1988). The data suggest that the Hawaiian Islands were colonized first from the western Pacific and from there colonized eastward through the Revillagigedo Islands and mainland Central America. From here, colonization probably spread south (through Costa Rica) and west to the Galápagos Islands, where we find the youngest clade in high frequency (III) (Fig. 4). The low haplotypic diversity and the presence of one dominant haplotype in Hawaii contrast with Galápagos and Revillagigedo that are characterized by high number of haplotypes. While there are caveats with attempts to reconstruct evolutionary history based on just a “snapshot” of present day diversity (see Karl et al. 2012), our findings suggest that colonization of Hawaii may have been by the first migrants from the western Pacific that established this population with CmP3 as the ancestral haplotype, but with CmP4.1 dominating in the eastern Pacific after subsequent colonization of Revillagigedo and the eastern Pacific mainland. Indeed, Revillagigedo is the only stock that shared haplotypes with all the other MUs in our study, further suggesting it provided a stepping-stone for radiation of green turtles from the Hawaiian rookeries into the eastern Pacific (Table 2, Fig. 4). Our results fit within a new paradigm for marine biodiversity articulated by Bowen et al. (2013), where oceanic islands, such as the Hawaiian and Revillagigedo Archipelagos, rather than being peripheral evolutionary graveyards, act as sources (as well as recipients) of marine biodiversity, providing a mechanism for further radiation in a biodiversity feedback model. On a regional scale, the Revillagigedo Islands appear to be a center of accumulation of shared haplotypes from Hawaii to the west, and from Galápagos and central America to the east, as well as a source of haplotypes to rookeries in the east, and possibly to Hawaii (Fig. 4). The markedly lower diversity in Hawaii relative to the eastern Pacific further suggests that colonization of these more remote and oceanographically isolated islands is older and unlikely to have been from more recent eastern Pacific sources. The low number of haplotypes found at this relatively large breeding population at FFS does not fit a model of a long-term stable population. This low diversity may be the result of continual population fluctuations, with rapid population declines and increases likely on the small atolls that characterize NW Hawaii, and CmP1.1 may not have been dominant in the past but instead the more central CmP3.1. Indeed, Frey et al. (2013) have suggested that recent increase in sporadic nesting in the main Hawaiian Islands represents new colonization by migrants from FFS and may provide a buffer to this population from impacts of sea level rise which threaten to inundate the current nesting atolls in NW Hawaii (Baker et al. 2006).

### Conservation implications

Our study shows that the major *C. mydas* nesting aggregations in the central and eastern Pacific form 5 distinct MUs for conservation purposes (Taylor and Dizon 1999; Palsboll et al. 2007; Waples and Gaggiotti 2006). This information provides a comprehensive baseline for improving MSA and assignment of individuals in fisheries bycatch and at foraging grounds (FG) to stocks for management purposes, particularly since we have been able to further differentiate the common CmP4 haplotype into informative haplotypes along with having identified many new haplotypes that are unique to specific MUs, so that ambiguous assignments of FG animals in past studies (e.g., Amorocho et al. 2012) can now be resolved.

Our results are consistent with previous reported mtDNA findings and reinforce the view that Hawaiian *C. mydas* represent a central North Pacific genetic stock (MU), that is, biologically and spatially distinct from other MUs in the Pacific (Dutton et al. 2008). The relatively small Revillagigedos nesting population has been largely overlooked, but is of special interest given the apparent historical role this MU has played in maintaining diversity in the region and connecting the central and eastern Pacific. It is important to protect these small, remote nesting sites, given the diversity of this rookery. Similarly the Costa Rica MU, while small relative to Michoacán and Galápagos, contains additional unique haplotypic diversity that warrants further attention. Our findings contribute to a new approach to managing sea turtles that recognizes the broader geographic units appropriate for maintaining the global diversity (see Taylor et al. 2010) and are consistent with the two *C. mydas* regional management units proposed for the central and eastern Pacific in Wallace et al. (2010). Further expansion of our study into a more comprehensive global phylogeographical analysis of *C. mydas* with data from the Indo-Pacific, Atlantic and Mediterranean is needed to inform future efforts to accurately identify subpopulations following IUCN criteria (Wallace et al. 2013), or distinct population segments (DPS) under the US Endangered Species Act (USFWS & NMFS 1996; Taylor et al. 2010) for this globally distributed threatened species. Our findings suggest that maintaining viable nesting habitat in the central Pacific rookeries in the Hawaiian Archipelago could be important for maintaining evolutionary potential and resilience of *C. mydas* on deeper time scales into the future.

## Data Accessibility

DNA sequences: GenBank accessions KC306643–KC306671, FJ917194, and KC771270.

## Appendix

**Table A1:** Mitochondrial DNA control region polymorphisms and haplotype designations for ca. 770-bp sequences from central and eastern Pacific *Chelonia mydas*. Shaded area marks the previously sequenced “short” 384-bp region. (•) have the same base as CmP1.1 and (–) are insertions or deletions. GenBank Accession numbers are indicated

	Base pair position																																						
Haplotype	49	50	115	134	208	209	212	228	229	250	270	335	336	404	408	409	411	419	469	506	507	508	509	510	511	512	513	588	622	647	648	651	659	664	669	671	704	733	Genbank ID
CmP1.1	–	–	C	A	G	A	T	A	T	A	A	G	T	G	A	T	G	A	T	–	–	–	–	–	–	–	–	C	G	T	A	A	G	A	C	G	A	T	KC306652
CmP2.1	–	–	•	•	A	•	C	•	•	G	•	•	•	•	•	•	•	–	•	–	–	–	–	–	–	–	–	•	•	•	•	•	A	•	•	•	•	•	KC306650
CmP3.1	–	–	•	•	•	•	C	•	•	•	•	•	•	•	•	•	•	•	•	–	–	–	–	–	–	–	–	•	•	•	•	•	•	•	•	•	•	•	KC306654
CmP3.2	–	–	•	•	•	•	C	•	•	•	•	•	•	•	•	•	•	•	•	–	–	–	–	–	–	–	–	•	•	•	•	G	•	•	•	•	•	•	KC306653
CmP4.1	–	–	•	•	•	•	C	•	•	•	•	•	•	•	•	•	•	•	C	–	–	–	–	–	–	–	–	•	•	•	•	•	•	•	•	•	•	•	KC306666
CmP4.2	–	–	A	•	•	•	C	•	•	•	•	•	•	•	•	•	•	•	C	–	–	–	–	–	–	–	–	•	•	•	•	•	•	•	•	•	•	•	KC306662
CmP4.3	–	–	•	G		•	C	•	•	•	•	•	•	•	•	•	•	•	C	–	–	–	–	–	–	–	–	•	•	•	•	•	•	•	T	•	•	•	KC306645
CmP4.4	–	–	•	•	•	•	C	•	•	•	•	•	•	•	•	•	•	•	C	–	–	–	–	–	–	–	–	•	A	•	•	•	•	•	•	•	•	•	KC306665
CmP4.5	–	–	•	•	•	•	C	•	•	•	•	•	•	•	•	•	•	•	C	–	–	–	–	–	–	–	–	•	•	C	•	•	•	•	•	•	•	•	KC306664
CmP4.6	–	–	•	•	•	•	C	•	•	•	•	•	•	•	•	•	•	•	C	–	–	–	–	–	–	–	–	•	•	•	•	•	•	•	T	•	•	•	KC306647
CmP4.7	–	–	•	•	•	•	C	•	•	•	•	•	•	•	•	•	•	•	C	–	–	–	–	–	–	–	–	•	•	•	•	•	•	•	•	A	•	•	KC306660
CmP4.8	–	–	•	•	•	•	C	•	•	•	•	•	•	•	•	•	•	•	C	–	–	–	–	–	–	–	–	•	•	•	•	•	•	•	•	•	G	•	KC306663
CmP4.9	–	–	•	•	•	•	C	•	•	•	•	•	•	•	•	•	•	•	C	–	–	–	–	–	–	–	–	•	•	•	•	•	•	•	T	•	•	C	KC306643
CmP4.11	–	–	•	•	•	•	C	•	•	•	•	•	•	•	•	•	•	•	C	–	–	–	–	–	–	–	–	•	•	•	–	•	A	•	•	A	•	•	KC306661
CmP5.1	–	–	•	•	•	•	C	•	•	G	•	•	C	•	•	•	•	•	•	–	–	–	–	–	–	–	–	•	•	•	•	•	•	•	•	•	•	•	KC306651
CmP6.1	–	–	•	•	•	•	C	•	•	•	•	•	•	•	G	•	•	•	•	–	–	–	–	–	–	–	–	•	•	•	•	•	•	•	•	•	•	•	KC306657
CmP6.2	–	–	•	•	•	•	C	•	•	•	•	•	•	•	G	•	•	•	•	–	–	–	–	–	–	–	–	T	•	•	•	•	•	•	•	•	•	•	KC306655
CmP7.1	–	–	•	•	•	•	C	•	•	•	•	A	•	•	•	•	•	•	C	–	–	–	–	–	–	–	–	•	•	•	•	•	•	•	•	•	•	•	KC306667
CmP8.1	–	–	•	•	•	G	C	•	•	•	•	•	•	•	•	•	•	•	•	–	–	–	–	–	–	–	–	•	•	•	•	•	•	•	•	•	•	•	KC306659
CmP8.2	–	–	•	•	•	G	C	•	•	•	•	•	•	•	•	•	•	•	•	–	–	–	–	–	–	–	–	•	•	•	•	•	•	G	•	•	•	•	KC306671
CmP8.3	A	A	•	•	•	G	C	•	•	•	•	•	•	•	•	•	•	•	•	–	–	–	–	–	–	–	–	•	•	•	•	•	•	•	•	•	•	•	KC771270
CmP9.1	–	–	•	•	•	•	C	G	•	•	•	A	•	•	•	•	•	•	C	–	–	–	–	–	–	–	–	•	•	•	•	•	•	•	•	•	•	•	KC306668
CmP11.1	–	–	•	•	•	•	C	•	•	•	•	A	•	•	G	•	•	•	•	–	–	–	–	–	–	–	–	•	•	•	•	•	•	•	•	•	•	•	KC306658
CmP12.1	–	–	•	•	•	•	C	•	•	•	•	•	•	•	•	C	•	•	C	–	–	–	–	–	–	–	–	•	•	•	•	•	•	•	T	•	•	•	KC306669
CmP13.1	–	–	•	•	•	•	C	•	•	•	G	•	•	•	•	•	•	•	C	–	–	–	–	–	–	–	–	•	•	•	•	•	•	•	T	•	•	•	KC306644
CmP14.1	–	–	•	•	•	•	C	•	C	•	•	•	•	•	G	•	•	•	•	–	–	–	–	–	–	–	–	•	•	•	•	•	•	•	•	•	•	•	KC306656
CmP15.1	–	–	•	•	•	G	C	•	•	•	•	•	•	•	•	•	•	•	C	–	–	–	–	–	–	–	–	•	•	•	•	•	•	•	T	•	•	•	KC306649
CmP17.1	–	–	•	•	•	•	C	•	•	•	•	•	•	•	•	•	A	•	C	–	–	–	–	–	–	–	–	•	•	•	•	•	•	•	T	•	•	•	KC306648
CmP17.2	–	–	•	•	•	•	C	•	•	•	•	•	•	•	•	•	A	•	C	–	–	–	–	–	–	–	–	•	•	•	•	•	•	•	•	•	•	•	KC306670
CmP24.1	–	–	•	•	•	•	C	•	•	•	•	•	•	A	•	•	•	•	C	–	–	–	–	–	–	–	–	•	•	•	•	•	•	•	T	•	•	•	KC306646
CmP93.1	–	–	•	•	•	•	C	•	•	•	•	•	•	•	•	•	•	•	C	C	A	A	T	G	T	T	G	•	•	•	•	•	•	•	T	•	•	•	FJ917195
